# Indents

**Published:** 1912-10-15

**Authors:** 


### INDENTS.

**☞Brief Articles of Technical or Scientific Value will receive notice and comment. Contributions invited.**

*The Last Straw.* Recently we have witnessed an encroachment upon medical practice which has mildly astonished us. We refer to the employment of nurses to act as physicians and in place of physicians on the part of the Department of Health. We are rapidly reaching the point where nothing will astonish us.

The use of nurses in this manner is practically a violation of the Medical Practice Act. But the attitude of the authorities is not one of "Well, what are you going to do about it," because no one feels obliged to assume that the medical profession is very keen about its own interests. Of course it is known that we will not do anything effective about it.

If the medical profession were the powerful body it ought to be, and could be, bureaucrats would not date to subject us to contumely of any kind.

We are sound asleep if we fancy that this act of the Health Department is the last imposition that we will suffer.

In our zeal for prophylaxis we ought to take some steps by way of anticipation of future acts of usurpation. Our professional maladies are threatening to advance and our powers of resistance are in sore need of some sort of vaccine treatment.—Arthur C. Jacobson in *Long Island Medical Journal.*

*The Next Dental Congress.* A dental congress may not, from a scientific or educational standpoint, be of much profit, yet it may; but there are other sides to the question involving sufficient interest and profit to justify the expenditure of time and money on such projects—the bringing together of men of prominence from all over the world, whom we, the masses, can see and admire, is one point. A gathering of this character will probably induce you and your class-mate, whom you have not seen since that glorious night following commencement exercises, to go; then comes the opportunity to renew old ties of friendship. The exhibitors, always a source of joy, gather in unusual numbers. As a rule the congress is held at the time of some exposition, which is of interest and value to us. These and many other inducements will be held out to us, when the next Dental Congress, which will be held at San Francisco in 1915, begins.

The Panama Exposition, to be held in celebration of the opening of the wonderful canal across the Isthmus of Panama, comes in 1915, and the dentists of California have held their preliminary meetings, organized and have two and one-half years to perfect the greatest Dental Congress ever known. They will wear their brains and bodies to remnants, spend their hard earned money, all for our pleasure and edification, while we step into palace cars, or take an ocean liner via the canal, and are their guests.—Editorial in *Western Dental Journal.*

*Practice of Medicine and Dentistry in the Canal Zone.* By an order which took effect December 14th, the President has decreed that it shall be unlawful for any person to practice or attempt to practice medicine, surgery, dentistry, pharmacy or midwifery within the Canal Zone without first having obtained a license therefor from the Board of Health of the Canal Zone. Any person thus offending shall be punished by a fine not exceeding $25, or by imprisonment in jail not exceeding thirty days; or by both such fine and imprisonment, in the discretion of the court; provided that this order shall not apply to commissioned surgeons of the United States Army and Navy or Marine Hospital Service, or to physicians, surgeons, dentists or pharmacists and their assistants and nurses employed by the

Isthmian Canal Commission, or to nurses acting under the orders of a licensed physician; and that any person shall be regarded as practicing medicine within the meaning of this order who shall prescribe for, operate on, or in any wise attempt to heal, cure or alleviate, or who shall in any wise treat any disease or any physical or mental ailment of another; provided that nothing in this order shall be construed to prohibit gratuitous services in case of emergency, or to the administering of ordinary household remedies.—*Journal American Medical Association.*

*Faith in the Dentists.* William Allen White has faith in the dentists of Emporia and publicly acknowledges it, although he allowed one small fly to light in the ointment by carelessly not blue-penciling the statement that *"No doubt this is a case of intelligent selfishness,"* for selfishness among the men who are active in this work is so rare it does not deserve mention.

Mr White says in the Emporia, Kansas, *Gazette*:

"The dentists of this town have asked the school board to provide a dental room in the new high school building. There the dentists propose to hold a dental clinic free. At the clinic the teeth of the children may be examined, and in necessary cases straightened, and temporary fillings put in. It is all to be free—without money or without price, to the community or to the children.

"No doubt this is a case of intelligent selfishness, but fundamentally it is a case of service—service primarily for the joy of it. Of course in the long run, to create the habit of giving attention to the teeth creates a demand for dentistry that will in the run of the years make dentistry more profitable. But that is remote. The fun of it, the fine part of it, the joy of it, is found in the fact that these men, turning aside at regular intervals from money-making, will be doing a real service to their fellows in this clinic. It's a fine thing for any community to have the spirit of service abroad in it."—*Western Dental Journal.*

*Pain.* Pain in the oral cavity, caused or increased by thermal change, indicates a pulp lesion. Pain in a tooth increased by pressure or percussion indicates a pericemental lesion. Dull, steady pain, not very severe, indicates vascular engorgement without infection. Steady, throbbing, sharp pain indicates infection. Accompanied by chills it indicates pus formation. Ulcerations in the oral cavity not accompanied by pain, chronic, sytemic trouble. Ulcerations of a markedly painful character point to local infection alone.—F. G. Worthley in *Western Journal.*

*Cutting Tools, A New Alloy.* An alloy said to be composed of cobalt 75 parts and chromium 25 parts has recently been introduced for the blades of pocket knives. It is almost as hard as mild steel, but much tougher. It rivals gold and platinum in its non-corrosive properties, and does not tarnish or rust under atmospheric influence of any kind whatsoever. Knife blades made of it remain bright after use on any kind of fruit. A little care is needed in sharpening; the blade is not laid flat on the stone, but at an angle of about 30 degrees, preferable upon a carborundum stone, carefully avoid making a wire edge. The final whetting is done on a strop coated with a thick layer of soap mixed with very fine carborundum. The keen edge obtained will last a long time with care.—*The Brass World.*

*Oral Hygiene.* The hygienic preservation of the mouth with the septic foci left in the mouth is of course to us an absurdity, and yet a careful investigation of what 90 per cent. of the dentists of this country consider this sort of treatment will show just such a condition of affairs. I am a very earnest advocate of mouth cleanliness among the poorest classes of children. I believe that if dental dispensary service to poor children were confined to this one feature much more benefit would ultimately accrue than by the use of questionable methods of dental repair and construction. I have seen much harm perpetrated and damage done to the human body by makeshift or cheap constructive dental service; nothing has tended more to produce unhygienic conditions. Operative dentistry in its true sense is more or less of a luxury; it is impossible to make it a necessity. We can teach oral hygiene in its real and true sense; but when it comes to the repair of the ravages of dental disease, anything that falls short of what should be done should be criticized as malpractice. It is not a benefit; it is a curse. That is the true picture of dentistry as performed by the mass of dentists for the people who cannot afford to pay for dentistry properly performed. The excuse that these dentists give for that kind of malpractice is that it is impossible for them to provide the proper kind of dentistry and earn a livelihood. My point has always been in this matter that the people whio cannot afford to pay for the preservation of the tooth so that it shall be preserved in an aseptic condition from further possible infection hereafter, would be far better off if they never had anything done to that individual tooth. It remains for a Section like this to establish some form of education that will bring the practice of stomatology out of such a condition. The one way is to make the general medical man understand what aseptic dentistry means and to be able to distinguish the difference between aseptic dentistry and septic dentistry. When the physician understands that that dentistry is taking away years of the life of that patient by the septic methods of work pursued, he is going to protest against that thing just the same as he would protest against some form of maltreatment of the patient's ears or eyes, or any other part of the body.—M. L. Rhein in *Journal A. M. A.*, July 13, 1912.

*Dangers of Prophylaxis of Children's Diseases.* A number of extensive and severe epidemics of measles happen to be prevailing just now at various points, and Mme. Nageotte queries whether the modern methods of prophylaxis of children's diseases are not breeding up trouble for the futuie. She says, "In childhood, measles, mumps and chicken-pox are mild and harmless as a rule, but when children are prevented from acquiring these diseases they grow up non-immune. The stream of infection is confined within artificial dikes but the day comes when the dike gives way at some point, and then the waters spread far and wide, carrying devastation to crowds of adult non-immunes. The prevailing epidemics among adults and children are the result of the efficiency of modern prophylaxis. The number of persons rendered immune by having had the disease has been reduced by artificial means, and the others are defenseless when the infection finds an opportunity to take its revenge." She thinks that the plan of keeping the children of a family out of school when one has measles, chicken-pox or mumps, is, to say the least, of extremely doubtful ultimate advantage.—*Journal A. M. A.*, June 22, 1912.

*How Often Should Infants be Nursed?* In an interesting communication in the *Jahrbuck fuer Kinderheilkunde* (noted in the *Wiener Med. Whochen.*, 1912, No. 20), Dr. Rietschel discusses the difficult problem of how long infants should receive the mother's breast. He concludes that, while nursing from five to six times in the day of twenty-four hours usually is justified, there are infants that require from seven to eight nursings in order to thrive well. In many instances the breasts become better developed by the greater frequency of emptying, and this may be an important factor in deciding a physician's instructions.

*Medal for Humanitarian Service.* The publishers of *American Medicine* have taken a step which deserves the commendation of every physician interested in his country's progress in medical science. They have dediced to award, annually, a gold medal to the American physician whose work during the preceding year can properly be considered of most value to humanity. The medal is not competitive—it is not a thing to be sought. It will be given only to the men whose work stands out beyond all others as of peculiar service to the world. The following directors have been selected to pass upon the qualifications of those deemed most worthy, and to select the man most deserving of recogition: Dr. William J. Robinson, New York, editor of *The Critic and Guide;* Dr. Claude L. Wheeler, editor of *The New York Medical Journal;* and Dr. H. Edwin Lewis, editor of *American Medicine.*

*Recovery of Swallowed Foreign Bodies.* The author gives an account of some recent experiences with oesophagoscopy and bronchoscopy. In one case a plate with teeth attached to it was found close below the constrictor muscle of the pharynx. It was seen, seized, and extracted by means of oesophagoscopy. Another woman who had swallowed a plate with teeth was subjected to oesophagoscopy, but one of the teeth broke off the plate, and it was found that further attempts to deliver the remainder of the foreign body caused bleeding. The plate was therefore cautiously seized and gradually pressed into the stomach, where it was left, the blades of the forceps being opened and moved backwards and forwards until clear of it. It was passed per anum after two days. A lady, aged 60 years, swallowed a plate with false teeth during an apoplexy. The plate was localized by means of X-rays. At a latei date oesophagoscopy was undertaken but the X-ray examination was not repeated. It was them found that the plate had left the oesophagus. It was seen in the pelvis a few days later, and ultimately passed naturally. A more difficult case was that of a man who swallowed a plate with teeth on August 10, 1910. This was said to have been seen and felt in the oesophagus. Later, while the X-rays showed a shadow in the situation indicated, nothing could be seen when the oesophagoscope was passed. The man is still under observation, since Glucksmann fears that this may be one of those rare cases of wandering of a foreign body. Cases are on record in which a foreign body has gained an entrance through a mucous surface aseptically and produced a variety of symptoms through its mechanical presence later on.—G. Glucksmann in *Berlin. Klin. Woch.*

